# Intravenous injection of beta-amyloid seeds promotes cerebral amyloid angiopathy (CAA)

**DOI:** 10.1186/s40478-018-0511-7

**Published:** 2018-03-05

**Authors:** Michael Burwinkel, Manuel Lutzenberger, Frank L. Heppner, Walter Schulz-Schaeffer, Michael Baier

**Affiliations:** 1 Proteinopathies/Neurodegenerative Diseases - ZBS6, Robert Koch-Institut, Berlin, Germany; 2 Department of Neuropathology, Charité - Universitätsmedizin Berlin, Berlin, Germany; 3 Department of Neuropathology, Saarland University, Homburg, Germany

## Abstract

Seeding and spread of beta-amyloid (Aβ) pathologies have been considered to be based on prion-like mechanisms. However, limited transmissibility of Aβ seeding activity upon peripheral exposure would represent a key difference to prions, not only in terms of pathogenesis but also in terms of potential transmission of disease. We partially characterized the seeded Aβ amyloidosis after intracerebral injection of various brain homogenates in APP/PS1 mice. One particularly seed-laden homogenate was selected to investigate the development of Aβ pathologies after intravenous exposure. We report here that a single intravenous injection of an Alzheimer disease patient’s-brain extract into APP/PS1 recipient mice led to cerebral amyloid angiopathy within 180 days post injection. Thus, vascular proteinopathies such as CAA are transmissible in mice via the intravenous route of peripheral exposure.

## Introduction

Intracerebral injections of beta-amyloid (Aβ) require femtogram quantities of brain-derived Aβ seeds to induce an Alzheimer’s disease (AD)-like pathology in amyloid precursor protein (APP)-transgenic APP23 or tg2576 mice [7, 14, 18, 19]. This process has been considered to be comparable to intracerebral infections with “classical” prions consisting of the misfolded prion protein (PrP^Sc^). The apparent ease of intracerebral transmission suggests that the seeding and spread of Aβ pathologies in brain tissue may - at least in part - occur in a manner similar to PrP^Sc^-based prions [2, 13]. Unlike prion transmissibility however, oral, intraocular, intranasal, and intravenous administration of Aβ seeds did not promote development of AD-like pathologies in APP23 transgenic mice. Only administrations of Aβ seeds via the intraperitoneal route were sufficient to induce a cerebral Aβ amyloidosis in this AD mouse model [8]. Interestingly, intravenous transmission of scrapie disease in mice is far more efficient than the intraperitoneal route of infection and is almost as potent as direct intracerebral injection [15]. Overall, limited transmissibility of Aβ seeding activity via peripheral exposure would represent a key difference to prions, not only in terms of pathogenesis but also in terms of potential transmission of disease.

To our knowledge the intravenous transmission of AD-like pathologies by Aβ seeds was so far only attempted in one experimental setting using transgenic APP23 mice as recipients and APP23-mouse brain-derived homogenate as Aβ seed-containing inoculates [6]. A separate study used synthetic Aβ peptides but the actual route of exposure was questionable [16]. To study Aβ seeding activity following intracerebral and intravenous administrations we decided to employ APP_Swe_/PS1dE9 mice (here termed APP/PS1 mice) as hosts.

## Methods

### Animals

The study was approved by the local animal welfare authority (Landesamt für Gesundheit und Soziales, Berlin, Germany). APP/PS1 mice backcrossed to C57BL/6 were obtained from The Jackson Laboratory. Further breeding was done by pairings with non-transgenic C57BL/6 wildtype mice to keep the line heterozygous.

### Brain tissue extracts

Mice brain extracts were derived from aged (15- to 18-month-old) APP/PS1 mice (termed APP) and age-matched non-transgenic, control C57Bl/6 mice (termed B6). Males and females were used but care was taken to keep gender ratios close to 50% (overall 52% males, 48% females). For the results presented in Fig. 2f the two small groups with *n* = 3 consisted of two males and one female each. Human brain extracts were derived from the frontal cortices of two 64 and 68 years old AD patients (termed AD1 and AD2) and from a 48-year-old non-demented control patient (termed HCT). Both AD patients were categorized as CERAD neuritic plaque score C and Braak tangle stage VI; the non-demented control was CERAD 0. Tissues were homogenized at 10% (wt/vol) in PBS, vortexed, sonicated for 5 s, and centrifuged at 3000 *g* for 5 min. Supernatant were then aliquoted and stored at − 70 °C. Western-blot analysis showed equal amounts of Aβ in patients AD1 and AD2 extracts, whereas no Aβ was detected in the HCT sample. The APP/PS1 mouse-derived extract contained at least 5 times more Aβ per ng total protein than the AD patients extracts (data not shown).

### Intracerebral and intravenous injections

Intracerebral injections (20 μl of 10% brain homogenates) were applied in the sagittal midline. Intravenous injections were performed by slowly applying a total volume of 60 μl of 10% brain extracts further diluted (1:3 *v*/v) in sterile isotonic saline into the tail veins.

### Tissue collection

Mice were killed with an overdose of isoflurane and transcardially perfused with ice-cold PBS. Subsequently, brains were removed and divided sagitally. Brains were fixed in paraformaldehyde (4% for 24 h and 2% for additional 2–14 days) at 4 °C followed by dehydration and embedding in paraffin. Time points of sacrifice were 360 days post injection for intracerebral challenge and 180 and 270 days post injection for the intravenous exposure.

### Histological studies

Aβ deposits were detected using anti-Abeta 4G8 antibody (SIG-39220; BioLegend) as described previously [17]. Double-stainings to confirm vascular amyloid deposition were done using the amyloid-specific luminescent-conjugated pentameric thiophen pFTAA [1] and the anti–alpha smooth muscle actin 1A4 antibody-Alexa Fluor 594 conjugate (ab202368, Abcam). The extent of the CAA was quantified by counting amyloid-positive blood vessels in thalami, cortices, as well as the cortically attached leptomeninges in at least 3 separate (120 μm apart) sections per mouse brain. The obtained numbers of Aβ-positive vessels per area were then taken for statistical analysis. Plaque loads were similarly determined in hippocampi and cortices.

### Statistical analysis

All data were analyzed using Prism 5 software (GraphPad Software Inc.). Statistical differences between groups were assessed using the two-tailed Mann-Whitney U test or, in case of small group sizes, the Kruskal-Wallis test and Dunn’s multiple comparison test.

## Results and discussion

To demonstrate the development of an Aβ amyloidosis triggered by intracerebral exposure to Aβ seeds in our system, we injected various brain homogenates intracerebrally into 6–8 weeks old APP/PS1 mice.

**Fig. 1:**
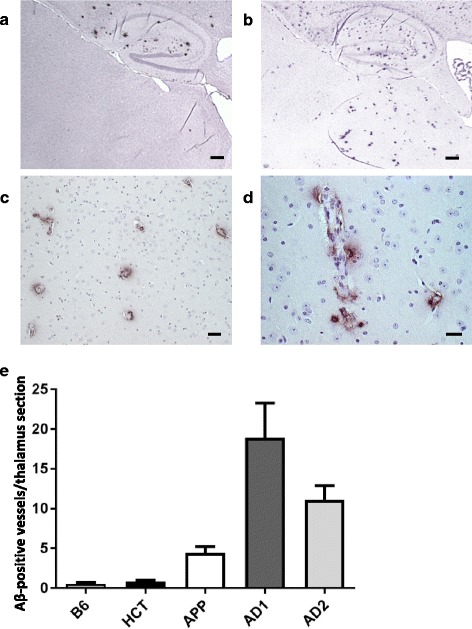
Vascular amyloid deposition following intracerebral injection of brain extracts into APP/PS1 mice. Aβ deposits were detected 360 days post injection using the 4G8 monoclonal antibody. **a** Representative overview of the hippocampus and thalamus regions upon injection of the negative control homogenate HCT. **b** Hippocampus and thalamus regions after injection of AD1 homogenate. Scale bar 500 μm. **c, d** Examples of thalamic CAA after injection of the AD1 extract at higher magnifications. The majority of Aβ deposits in the thalamus is vascular. Scale bars 25 μm in (**c**) and 12.5 μm in (**d**). **e** Quantification of thalamic CAA 360 days after intracerebral injection of brain extracts. Indicated is the mean ± SEM. Mann-Whitney U test, group sizes *n* = 5 (HCT), *n* = 6 (B6), n = 6 (APP), *n* = 7 (AD1), and *n* = 7 (AD2). *P* = 0.003 for B6 versus AD1 and AD2; *p* = 0.0085 B6 versus APP, *p* = 0.38 B6 versus HCT, *p* = 0.1026 for AD1 versus AD2. Of note, the CAA in AD patients extracts AD1- and AD2-injected mice was significantly more pronounced than in mice receiving the APP/PS1 brain homogenate; *p* = 0.0264 and *p* = 0.0052 for AD1 and AD2 versus APP, respectively

**Fig. 2:**
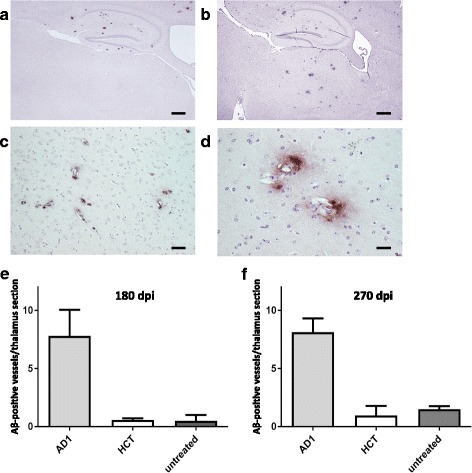
CAA following intravenous injection of brain extracts into APP/PS1 mice. **a** Representative overview of the hippocampus and thalamus regions upon injection of the negative control homogenate HCT. **b** Hippocampus and thalamus regions after injection of AD patient homogenate AD1, 180 days post injection. Scale bar 500 μm. **c** Example of thalamic CAA after injection of the AD1 extract at higher magnification. The majority of Aβ deposits in the thalamus is vascular. Scale bar 25 μm. **d** Thalamic CAA with notable spread of Aβ deposition into the adjacent tissue after injection of the AD1 extract. Scale bar 12.5 μm. **e** Quantification of thalamic CAA 180 days after intravenous injection of brain extracts. Untreated APP/PS1 mice of the same age, which did not receive any injections, were included for additional comparison. Indicated is the mean ± SEM. Mann-Whitney U test, group sizes *n* = 6 (AD1), *n* = 6 (HCT) and *n* = 7 (untreated). *P* = 0.008 and *p* = 0.0062 for AD1 versus HCT and the untreated group of mice, respectively. **f** Quantification of thalamic CAA 270 days after intravenous injection of brain extracts. Indicated is the mean ± SEM. Kruskal-Wallis test, group sizes *n* = 3 (AD1), *n* = 3 (HCT) and *n* = 6 (untreated). *P* = 0.038, with *p* < 0.1 for AD1 versus HCT and the untreated group, respectively, and *p* > 0.1 for HCT versus untreated (Dunn’s multiple comparison test)

Three hundred sixty days post intracerebral injection cortices and hippocampi of all APP/PS1 mice were, as expected for this mouse line at an age of 13.5–14 months, loaded with amyloid plaques regardless of the source of the injected brain homogenate (data not shown). However, in particular the AD1- and AD2-injected mice showed a pronounced vascular amyloid deposition affecting multiple small vessels in the thalamus region, which was not seen upon injection of the negative control extracts (Fig. 1). Of note, beginning at an age of about 6 months, APP/PS1 mice develop a progressive cerebral amyloid angiopathy typically assigned to the leptomeninges, while CAA in the thalamic region has not been described so far [9]. This is in line with our findings in untreated APP/PS1 mice, in which up to an age of 14 months thalamic CAA is not a prominent feature (Fig. 1e and Fig. 2e, f).

**Fig. 3:**
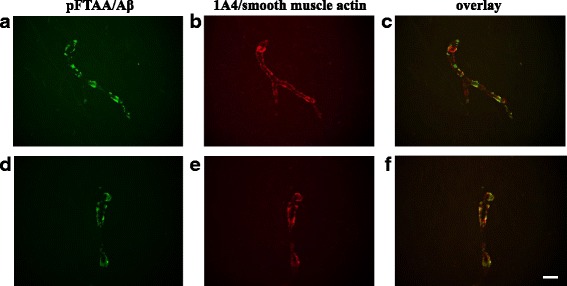
Double-stainings with amyloid-binding compound pFTAA and anti-smooth muscle actin antibody 1A4 to demonstrate the vascular Aβ deposition in thalami of intravenously AD1 injected mice. **a**, **d** staining with pFTAA; **b**, **e** staining of the same section with 1A4 antibody; **c**, **f** overlay of the pFTAA (green)/1A4 (red) stains. Scale bar 12.5 mm

To test transmissibility of an Aβ amyloidosis following intravenous exposure single injections of diluted brain extracts AD1 and HCT into the tail veins of 6–8 weeks old APP/PS1 mice were carried out. The first time point of analysis was 180 dpi when the mice were 7.5–8 months old. Most strikingly, a significantly higher number of Aβ-decorated blood vessels was evident in the thalamus areas of the AD1 group in comparison to control-injected and to untreated mice (Fig. 2). Essentially the same observations were made at a later time point, namely at 270 dpi. Just like before, the thalamic CAA was clearly more pronounced in the AD1-injected group compared to the controls (Fig. 2f). Double-stainings with amyloid-binding compound pFTAA and anti-smooth muscle actin antibody 1A4 additionally confirmed the deposition of Aβ in the thalamic vasculature (Fig. 3).

**Fig. 4:**
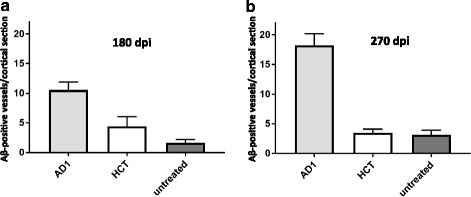
CAA in cortices and cortically attached leptomeninges following intravenous injection of brain extracts into APP/PS1 mice. **a** CAA quantification 180 days after intravenous injection of brain extracts. Indicated is the mean ± SEM. Mann-Whitney U test, group sizes as in Fig. 2e. *P* = 0.027 and *p* = 0.016 for AD1 versus HCT and the untreated group of mice, respectively. **b** CAA quantification 270 days after intravenous injection of brain extracts. Kruskal-Wallis test, group sizes as in Fig. 2f. *P* = 0.0214, with *p* < 0.05 for AD1 versus the untreated group, and *p* > 0.1 for the other comparisons (Dunn’s multiple comparison test)

Furthermore, we also observed significant increased CAA in cortices and attached leptomeninges upon intravenous injection of the AD1 extract compared to the controls (Fig. 4). However, at both time points, 180 and 270 dpi, neither hippocampal nor cortical amyloid plaque loads seemed to be affected by the intravenously applied exposure to Aβ seeds (Table 1).

**Table 1:** Hippocampal and cortical amyloid plaque counts after intravenous injections^a^

	180 dpi	270 dpi
Hippocampus		
AD1	9.0 +/− 0.9	29.0 +/− 6.4
HCT	9.3 +/− 2.1	22.1 +/− 8.6
untreated	9.7 +/− 3.1	30.0 +/− 6.0
Cortex		
AD1	31.7 +/− 4.0	116.0 +/− 21.5
HCT	35,6 +/− 7.5	82 +/− 19.3
untreated	29.4 +/− 9.0	86.2 +/− 11.5

^a^ Group sizes as in Fig. 2e (180 dpi) and f (270 dpi). Differences between groups for a given time point are not significant (*p* > 0.1 in all comparisons)

Our results demonstrate for the first time that intravenously administered Aβ seeds of human origin promote vascular Aβ deposition in APP/PS1 mice. In APP/PS1 mice the transgenes are, due to the employed *Prnp* promoter, also expressed in the periphery, which is not the case in APP23 mice, in which the expression of the transgene is confined to neurons of the CNS [11, 22]. Thus, in APP/PS1 mice interaction of injected Aβ seeds with already present peripheral human Aβ may support the transport of seeds towards the brain.

Interestingly, the amyloidosis occurring after intraperitoneal administration of Aβ seeds was also seen to be predominantly associated with blood vessels [8]. Of note, the AD patient-derived brain extracts triggered a considerably more pronounced thalamic CAA than the APP/PS1 mouse brain-derived inoculum (Fig. 1e). “Strain”-like properties of Aβ seeds or Aβ aggregates have been suggested to play a role in the pathogenesis of distinct AD phenotypes [4, 10, 20]. Likewise, Aβ pathologies may vary upon transmission depending on the characteristics of the transmitted seeds and, possibly, the route of exposure.

In summary, we report here that intravenous injection of Aβ seeds derived from human AD brain extract promoted a vascular Aβ amyloidosis in APP/PS1 mice. Our data add novel aspects to the ongoing discussion on the transmissibility of proteinopathies such as prion diseases and AD [13]. There are to date no data allowing to conclude that Aβ seeding activity is transmissible via blood or blood products [5]. However, at least in mice blood-derived Aβ can pass the blood-brain barrier to form amyloid deposits in the brain [3]. Moreover, observational evidence suggests that Aβ pathologies were transmitted by repeated intramuscular injections of cadaveric-derived, human growth hormone preparations [12, 21]. Thus, further research is required to identify the factors contributing to transmissibility of misfolded proteins and ultimately to prevent transmission of amyloid pathologies in humans.
